# A rare case of a peritoneal cyst arising from the falciform ligament

**DOI:** 10.1186/1757-1626-2-134

**Published:** 2009-02-09

**Authors:** Anish Patel, Valentina Lefemine, Bangalore S Ramanand

**Affiliations:** 1 Department of General Surgery, Glan Clwyd Hospital, Rhyl, Denbighshire, LL18 5UJ, UK

## Abstract

Peritoneal cysts are rare benign intra-abdominal lesions most commonly found attached to the mesentery of the ileum. They may be asymptomatic, present with abdominal pain, bloating or discovered incidentally. We describe the case of a peritoneal cyst located in an unusual position in a female patient; attached to the falciform ligament.

## Case presentation

**Figure 1:**
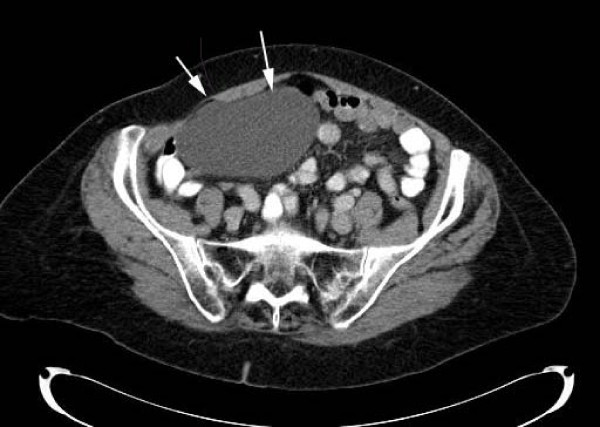
**Abdominal CT scan**. (Arrows indicate boundaries of cyst).

We present a case of a 61 year old lady who presented with abdominal pain and bloating of 12 months duration. Her past medical history was unremarkable. Examination revealed mild abdominal distension with a sense of fullness in the right flank. There was mild tenderness to deep palpation. Her full blood count, renal and liver function tests were normal. CT scanning confirmed a large cystic structure in the right side of her abdomen (Figure 1). Initially she was managed conservatively however due to persisting symptoms she was taken for surgical excision.

**Figure 2:**
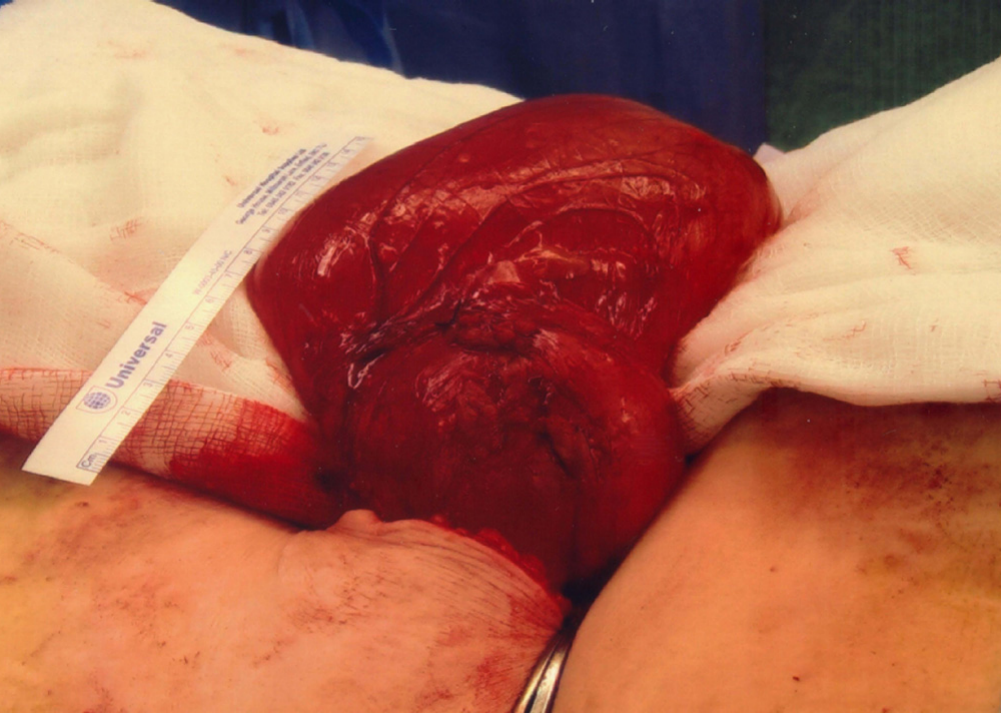
**Operative findings: Large cyst arising from the falciform ligament**.

At laparotomy, a 16 × 10 cm thin walled cyst was found attached to the falciform ligament (Figure 2). It contained gelatinous substance. The cyst was completely excised. She made good progress after her operation with complete resolution of her symptoms and she remains well 6 months after surgery.

## Discussion

Peritoneal cysts are rare, usually benign tumours that are cystic in nature. They can be located anywhere in the mesentery between the duodenum to the rectum although most are found in the ileal mesentery. The estimated incidence of these lesions is 1:100,000 and 1:20,000 in adults and children respectively [1,2]. Of the 900 or so published cases only four were found to be malignant [3]. They appear to have no sex predilection. Peritoneal cysts can been classified according to the de Perrot classification [1] based on their cellular origin (1 lymphatic, 2-Mesothelial, 3-enteric, 4-Urogenital, 5-Mature cystic teratoma, 6-Non pancreatic pseudocysts). What makes this particular case interesting is that it arose from the falciform ligament. Such lesions are much rarer with only 12 cases described in the English literature since the first report in 1909 [4].

Peritoneal cysts were first described by Benevenni, an Italian anatomist during a post mortem examination in 1507. In 1880 Tillaux performed the first successful surgical excision of a peritoneal cyst.

Most peritoneal cysts are asymptomatic. Symptoms arise due to the pressure effects exerted by these lesions onto surrounding structures. Commonly these include abdominal pain, distension, constipation and vomiting. Common complications include, rupture, obstruction, infection, torsion or haemorrhage within the cyst [5-8]

The diagnosis of such lesions requires a thorough history, clinical examination and a high index of suspicion coupled with radiographic imaging. The most useful imaging modalities are abdominal ultrasound and computed tomography [9].

Treatment of cysts should be considered in patients who develop symptoms or when cysts cause complications. Treatment options include percutaneous drainage, complete excision or marsupialisation. If bowel is involved, a segmental resection of the affected bowel may need to be performed. Complete excision should be considered the preferred treatment as it prevents recurrence. Partial excision or marsupialisation can result in infection and sinuses and therefore should be reserved for patients whose cysts involve a large proportion of the bowel mesentery. Simple drainage usually results in recurrence of the cyst and should not be performed.

Laparoscopic complete excision has been shown to have the added advantage of a shorter post operative course and fewer complications [3] and should be considered the gold standard of treatment. In our case laparoscopic surgery was not possible due to the size and position of the cyst, it therefore applies to appropriately selected patients.

## Consent

Written informed consent was obtained from the patient for publication of this case report and accompanying images. A copy of the written consent is available for review by the Editor-in-Chief of this journal.

## Competing interests

The authors declare that they have no competing interests.

## Authors' contributions

AP and VL co wrote and edited the manuscript. BSR performed the surgical excision.

## References

[B1] de Perrot M Bründler M Tötsch M Mentha G Morel P Mesenteric cysts. Toward less confusion? Dig Surg 2000 17 4 323 8 10.1159/000018872 11053936

[B2] Kwan E Lau H Yuen WK Laparascopic resection of a mesenteric cyst Gastrointest Endosc 2004 59 1 154 156 10.1016/S0016-5107(03)02365-4 14722577

[B3] Sahin DA Akbulut G Saykol V San O Tokyol C Dilek ON Laparoscopic enucleation of mesenteric cyst: a case report Mt Sinai J Med 2006 73 7 1019 20 17195889

[B4] Bryan DH Pillarisetty S Cyst of the falciform ligament of the liver: a rare cause of right upper quadrant pain Am Surg 1992 58 12 779 8 1456607

[B5] Namasivayam J Ziervogel MA Hollman AS Case report: volvulus of a mesenteric cyst – an unusual complication diagnosed by CT Clin Radiol 1992 46 3 211 2 10.1016/S0009-9260(05)80450-7 1395431

[B6] Iuchtman M Soimu U Amar M Peritonitis caused by a ruptured infected mesenteric cyst J Clin Gastroenterol 2001 32 452 3 10.1097/00004836-200105000-00022 11319324

[B7] Takeuchi K Takaya Y Maeda K Maruo T Peritonitis caused by a ruptured, infected mesenteric cyst initially interpreted as an ovarian cyst. A case report J Reprod Med 2004 49 1 65 7 14976800

[B8] Barut I Tarhan OR Ciris M Akdeniz Y Bulbul M Intestinal obstruction due to a mesenteric cyst Yonsei Med J 2004 45 2 356 8 15119014 10.3349/ymj.2004.45.2.356

[B9] Liew SCC Glenn DC Storey DW Mesenteric Cyst Aust N Z J Surg 1994 64 741 744 10.1111/j.1445-2197.1994.tb04530.x 7945079

